# “Evaluating the Implementation and Best Practices of Foster Care in Greece: Insights from Law 4538/2018 and the Anynet System”

**DOI:** 10.1192/j.eurpsy.2025.1178

**Published:** 2025-08-26

**Authors:** I. Farmakopoulou, V. Baltsioti, M. Theodoratou

**Affiliations:** 1 Sciences of Education and Social Work, University of Patras; 2 Social Sciences, Hellenic Open University, Patras, Greece; 3 Psychology, Neapolis University Pafos, Pafos, Cyprus

## Abstract

**Introduction:**

The institution of child fostering aims to protect minors who lack a secure family environment. In Greece, the implementation of Law 4538/2018 and the Anynet electronic system sought to de-institutionalize minors. This paper, based on a doctoral thesis, investigates the law’s impact and identifies best practices in child fostering.

**Objectives:**

This study aims to evaluate the implementation of Law 4538/2018 in Greece and the Anynet electronic system, focusing on their impact on de-institutionalizing minors. Additionally, it seeks to identify best practices in the foster care system and their application in child protection settings.

**Methods:**

A mixed-method approach was used. The first part involved a quantitative study targeting Directors of Child Protection Frameworks across Greece and foster parents. The second part employed a qualitative case study method, using semi-structured interviews with social workers from four selected Child Protection Frameworks recognized as examples of best practices

**Results:**

The research revealed that, despite the introduction of Law 4538/2018, foster care in Greece remains underutilized, especially for adolescents. Long-term fostering is the most common form, with most children aged four to six. Contact with biological parents is limited, often leading to adoption. Social workers lack sufficient training and familiarity with Anynet, impacting foster placements. However, child protection frameworks that applied specialized strategies for difficult cases saw fewer placement failures. A Unified Foster Care Protocol could standardize and improve foster care practices nationwide.

**Conclusions:**

Though Law 4538/2018 and Anynet are steps forward, Greece’s foster care system is still underdeveloped, with minimal increases in placements. Targeted case management and better social worker training are essential for success. Implementing a Unified Foster Care Protocol could enhance consistency and improve outcomes for fostered children.

**Disclosure of Interest:**

None Declared

